# Supplementary material for: A group resilience training program for people with multiple sclerosis: Study protocol of a multi-centre cluster-randomized controlled trial (multi-READY for MS)
Source: PLoS One. 2022 May 2;17(5):e0267245. doi: 10.1371/journal.pone.0267245 (PMC9060330; doi:10.1371/journal.pone.0267245)
Supplement: S2 Appendix — (DOCX) [file pone.0267245.s002.docx]

**S2 APPENDIX - READY Sessions**

(This material is provided by Prof. Kenneth Pakenham, Faculty of Psychology, University of Queensland.)

**Session 1: Introduction to the READY program**

The introduction session aims to: (1) build rapport; (2) outline the structure, purpose, and theoretical orientation of the READY Program and the READY personal plan; and (3) educate around protective factors for resilience and early warning signs of low resilience.

Session 1 introduces participants and facilitator and establishes group ground rules (e.g., participants can withdraw at any time, all information is confidential).

The READY program is outlined and each session discussed in terms of aims, main strategies used, and desired outcomes. The program content entails psychoeducation on resilience, examines the READY model of resilience, and outlines the protective factors for resilience (i.e., cognitive flexibility, meaning, social connectedness, coping strategies, and acceptance). Participants are encouraged to reflect on general and personal signs of low resilience and note these in their READY personal plan. The content of Session 1 is then reviewed prior to conclusion of the session.

**Session 2: Mindfulness**

The aims of the mindfulness session are to: (1) review the previous session and READY personal plan activities; (2) review the READY resilience model; (3) introduce mindfulness; and (4) practise mindfulness exercises. Session 2 begins with a review of Session 1 to reinforce concepts of resilience and protective factors. Psychoeducation is conducted on mindfulness and its importance in resilience, and compared to the unhelpful role of ‘mindlessness’ in daily stress. A variety of mindfulness exercises are practised including mindfulness of eating a sultana, mindfulness of sound and sight, mindfulness of breathing, and mindfulness of physical sensations. Participants are encouraged to share their experiences within the group setting following each exercise. Prior to the conclusion of Session 2, participants are given formal and informal mindfulness exercises to practise between sessions and incorporate into their READY personal plan.

**Session 3: Acceptance**

The aims of the acceptance session are to: (1) review the previous session and READY personal plan activities; (2) review the READY resilience model; (3) educate participants on emotions and emotion management strategies; (4) educate participants on the concept of acceptance; and (5) practise acceptance strategies. Session 5 begins with a review of the content delivered during Session 2 and a mindfulness exercise. Education and discussion are conducted in regard to emotion, experiential avoidance, and behavioural and cognitive methods for avoidance.

Acceptance (allowing thoughts to exist and acknowledging the discomfort without struggle) is presented as an alternative strategy to manage uncomfortable emotions. Various experiential acceptance exercises are delivered during the session (e.g., the “Stop; Notice the unwanted feeling, thought, bodily sensation, memory, or image; Let go of the struggle; Make space for it” strategy) are practised. As with previous sessions, participants are encouraged to share their experiences among the group following experiential practice of each exercise. Prior to the conclusion of Session 3, participants are encouraged to reflect on their emotional learning and practise acceptance strategies daily, recording their experiences in their READY personal plan.

**Session 4: Defusion I**

The aims of the first defusion session are to: (1) review the previous session, READY personal plan activities, and READY resilience model; (2) educate participants on fusion and defusion from thoughts; (3) teach participants to identify unhelpful thoughts; and (4) practise defusion strategies. Session 4 begins with a mindfulness exercise and a review of the content delivered in Session 2, including participant progress with the mindfulness strategies delivered during that session. Education is conducted on the differences between thought fusion and defusion, and a variety of defusion strategies are delivered with participants encouraged to practice these during the session and review their experience within the group. Participants are asked to identify unhelpful thoughts, practise formal and real-time defusion, and keep a record of their practise between sessions. Prior to the conclusion of Session 4, participants are given formal and informal defusion exercises to practise between sessions and incorporate into their READY personal plan.

**Session 5: Defusion II and the Observer Self**

The aim of the second defusion session includes the following: (1) review the previous session, READY personal plan activities, and READY resilience model; (2) trouble shoot defusion strategies learnt in Session 4; (3) practise additional defusion strategies; (4) educate participants on the “observer’ self”; and (5) help participants identify unhelpful stories about the self. Session 4 begins with a mindfulness exercise and a review of the content delivered in Session 4 to explore the progress that participants had made with defusion over the previous week. The session focuses on troubleshooting any difficulties that participants identified in practicing the defusion strategies already delivered prior to the introduction of additional defusion strategies.

Participants are encouraged to practise the additional defusion techniques during the session, and then review their experience with the group. Psychoeducation is delivered regarding the concept of the ‘Observer Self’ in contrast to the ‘Conceptualised Self’.

Participants reflect upon the thoughts, images, and memories that substantiate their conceptualised self (i.e., personal stories), explor the impact of changing them, and consider the potential of adopting/ modifying new stories of themselves in the context of living with a diagnosis of MS. Prior to the conclusion of Session 5, participants are encouraged to utilise their READY personal plan to reflect further on their ‘stories’ and continue to practice the defusion strategies delivered during the session.

**Session 6: Values and Meaningful Action**

The aims of the values and committed action session are to: (1) review the previous session, READY personal plan activities, and READY resilience model; (2) educate participants on values; (3) assist participants to develop a value statement; and (4) assist participants to develop meaningful action consistent with their values; (5) educate participants on social connectedness and resilience, types of useful social support responses, and identify barriers to participating in social support; and (6) explore self-care strategies to promote resilience. Session 6 began with a mindfulness exercise and a review of the content delivered in Session 5, including the progress participants had made in their practice of the acceptance and defusion strategies over the previous week.

The importance of personal values and meaningful action are discussed, including the difference between values, goals, and feelings. Participants are encouraged to examine their own personal values and ideal behaviours across various life domains (i.e., family, intimate relationships, and health), develop a values statement, review the consistency between their actions and values, and develop a new, values-consistent action. Prior to the conclusion of Session 6, participants are encouraged to implement the meaningful action they identified during the session over the coming week, as well as identify another value and meaningful action and incorporate this into their READY personal plan.

**Session 7: Finale and Future Planning**

The aim of the final session is to: (1) review the previous session, READY personal plan activities, and READY resilience model; (2) understand the links between life domains, protective factors, and strategies to build resilience; (3) identify and demonstrate strategies to promote meaning, social support and connectedness, and relaxation; (4) identify resilient and non-resilient traits in relationships with others, meaning, and doing; (5) identify potential barriers to implementing resilience strategies, and ways to resolve these; and (6) refine a personal plan to identify and address early warning signs of low resilience. Session 7 begins with a mindfulness exercise and a review of the content delivered in Session 6. Discussion is held around participant progress with regard to their identification of personal values and implementation of meaningful action. The session content focused on reviewing all important aspects of the program, synthesising key learnings, and ensuring participants had an applied understanding of the skills delivered throughout the intervention. The main areas reviewed included the characteristics of resilience and non-resilience, protective factors for resilience, meaning, social connectedness, coping strategies, cognitive flexibility, and acceptance. Resilience building strategies are reviewed and mapped onto the key protective factors. The group ends with the facilitator thanking all participants for their engagement, and asking them to each discuss one or two things they are going to take away from the program.

**Booster session** (approximately 5 weeks following Session 7)**:**

The booster session commences with a mindfulness exercise and reviews the content covered across the READY program. Participants are encouraged to share their progress and experience of applying the strategies and techniques learned through attending the READY program.
