# Supplementary material for: A group resilience training program for people with multiple sclerosis: Study protocol of a multi-centre cluster-randomized controlled trial (multi-READY for MS)
Source: PLoS One. 2022 May 2;17(5):e0267245. doi: 10.1371/journal.pone.0267245 (PMC9060330; doi:10.1371/journal.pone.0267245)
Supplement: S3 Appendix — (DOCX) [file pone.0267245.s003.docx]

**S3 APPENDIX - Relaxation Sessions**

**Session 1: Introduction to the Relaxation program**

The introduction session aims to: (1) build rapport; (2) outline the structure, purpose, and theoretical orientation of the Relaxation program and the home practice; and (3) introduce some practices (fractioned relaxation; arms weight exercise). Participants will have group discussion, and will be given formal relaxation exercises (and audio) to practise between sessions.

**Session 2**

The aims of the 2^nd^ session are to: (1) review the previous session and the home practice; (2) consolidate some relaxation exercises (fractioned relaxation; arms weight exercise); and (3) introduce new ones (legs weight exercise; whole body weight exercise). Participants will have group discussion, and will be given formal relaxation exercises (and audio) to practise between sessions.

**Session 3**

The aims of the 3^rd^ session are to: (1) review the previous session and the home practice; (2) consolidate some relaxation exercises (fractioned relaxation; whole body weight exercise); and (3) introduce new ones (arm warmth exercise). Participants will have group discussion, and will be given formal relaxation exercises (and audio) to practise between sessions.

**Session 4**

The aims of the 4^th^ session are to: (1) review the previous session and the home practice; (2) consolidate some relaxation exercises (fractioned relaxation); and (3) introduce new ones (whole body weight and warmth exercise). Participants will have group discussion, and will be given formal relaxation exercises (and audio) to practise between sessions.

**Session 5**

The aims of the 5th session are to: (1) review the previous session and the home practice; (2) consolidate some relaxation exercises (fractioned relaxation; whole body weight and warmth exercise); and (3) introduce new ones (breathing exercise). Participants will have group discussion, and will be given formal relaxation exercises (and audio) to practise between sessions.

**Session 6**

The aims of the 6^th^ session are to: (1) review the previous session and the home practice; (2) consolidate some relaxation exercises (fractioned relaxation; weight, warmth, breathing exercise); and (3) introduce new ones (heart exercise). Participants will have group discussion, and will be given formal relaxation exercises (and audio) to practise between sessions.

**Session 7**

The aims of the last session are to: (1) review the previous session and the home practice; (2) consolidate some relaxation exercises (fractioned relaxation; weight, warmth, breathing, hearth exercise); and (3) introduce new ones (plexus exercise; fresh forehead exercise). Participants will have group discussion, and will be given formal relaxation exercises (and audio) to practise between sessions.

**Booster session** (approximately 5 weeks following Session 7)**:**

The aims of the last session are to: (1) review the program and the home practice; (2) consolidate relaxation exercises. Participants will be encouraged to share their progress and experience of applying relaxation to their daily life.
