# Supplementary material for: A group resilience training program for people with multiple sclerosis: Study protocol of a multi-centre cluster-randomized controlled trial (multi-READY for MS)
Source: PLoS One. 2022 May 2;17(5):e0267245. doi: 10.1371/journal.pone.0267245 (PMC9060330; doi:10.1371/journal.pone.0267245)
Supplement: S4 Appendix — (DOCX) [file pone.0267245.s004.docx]

**S4 Appendix – READY for MS Session Fidelity Checklist**

**An example**

**READY for MS - Session Checklist 1**

Location:

Facilitator:

Date:

Time and duration:

Attendance:

*Please, indicate in the checklist above if each of the activities reported below have been done. Write Yes if the activity was run as reported in the Session Manual, No, and reasons why, If it was skipped or done differently.*

Clinical Notes

| **Activity done (Yes/No)** | **Contents** |
| --- | --- |
|  | 1. Welcome & housekeeping |
|  | 1. Meet group members - Icebreaker” |
|  | 1. Guidelines for group work |
|  | 1. What is Resilience? |
|  | - 1. Group Discussion |
|  | - 1. Didactic teaching |
|  | 1. *READY* Model of Resilience |
|  | 1. Warning Signs of Low Resilience |
|  | - 1. Didactic teaching |
|  | - 1. Group discussion |
|  | 1. *READY* program overview |
|  | - 1. Didactic teaching |
|  | - 1. Group discussion |
|  | 1. The *READY* Resources – book and personal plan |
|  | 1. Key Learning |
|  | 1. READY Personal Plan in session |
|  | 1. Homework and session close |
