# Supplementary material for: A group resilience training program for people with multiple sclerosis: Study protocol of a multi-centre cluster-randomized controlled trial (multi-READY for MS)
Source: PLoS One. 2022 May 2;17(5):e0267245. doi: 10.1371/journal.pone.0267245 (PMC9060330; doi:10.1371/journal.pone.0267245)
Supplement: S5 Appendix — (DOCX) [file pone.0267245.s005.docx]

**S5 Appendix – Relaxation Session Fidelity Checklist**

**An example**

**Relaxation - Session Checklist 1**

Location:

Facilitator:

Date:

Time and duration:

Attendance:

*Please, indicate in the checklist above if each of the activities reported below have been done. Write Yes if the activity was run as reported in the Session Manual, No, and reasons why, If it was skipped or done differently.*

| **Activity done (Yes/No)** | **Contents** |
| --- | --- |
|  | 1. Welcome & housekeeping |
|  | 1. Brief presentation of the facilitator |
|  | 1. Meet group members – Icebreaker |
|  | 1. Overview of the training |
|  | 1. What is relaxation? |
|  | 1. What is autogenic training? |
|  | 1. Exercise 1 – Fractional relaxation |
|  | 1. Exercise 2 – Arms weight |
|  | 1. Homework and session close |

Clinical Notes
