# Supplementary material for: A group resilience training program for people with multiple sclerosis: Study protocol of a multi-centre cluster-randomized controlled trial (multi-READY for MS)
Source: PLoS One. 2022 May 2;17(5):e0267245. doi: 10.1371/journal.pone.0267245 (PMC9060330; doi:10.1371/journal.pone.0267245)
Supplement: S6 Appendix — (DOCX) [file pone.0267245.s006.docx]

**S5 Appendix – Drop-out Scenarios**

**Scenario 1**

Assuming a drop-out of 20% uniformly distributed among clusters (i.e. two individuals lost within each cluster): 12 clusters per treatment arm (total number of clusters=24) with eight individuals per cluster (total sample size 192) achieves 92.1% power to detect a mean difference of 9.66 between the two arms in terms of change in CD-RISC 25 at 3-month follow-up (Donner A, Klar, 1996). The SD of change in the CD-RISC 25 at 3-month is assumed equal to 14.53. The ICC is assumed, conservatively, equal to 0.10. The type I error (alpha) is assumed to 0.05.

**Scenario 2**

We assumed a drop-out of 20% (e.g. 5% failure to reach the enrollment target of 10 participants per group and 15% drop-out during intervention/follow-up) among clusters (i.e. 0 individuals lost within two clusters, one individual lost within five clusters, two individuals lost within eight clusters, three individuals lost within nine clusters). Since no exact formula exists for such a scenario, we performed a simulation study. We simulated 24 (number of clusters) times ten observations (subjects) from a normal distribution with mean equal to 9.66, SD equal to 14.53 and ICC=0.10. Then we randomly selected five clusters where we randomly deleted one subject, we randomly selected eight clusters where we randomly deleted two subjects each, we randomly selected nine clusters where we randomly deleted three subjects each. We simulated 1000 samples and computed the observed statistical power. Twelve clusters per treatment arm (total number of clusters=24) with 10 individuals in two clusters, nine individuals in five clusters, eight individuals in eight clusters, and seven individuals in the remaining nine clusters (total sample size 192) achieves 92.0% power to detect a mean difference of 9.66 between the two arms in terms of change in CD-RISC 25 at 3-month follow-up (Donner A, Klar, 1996).

**Scenario 3**

We assumed a variables drop-out of 25% (e.g. 10% failure to reach the enrollment target of 10 participants per group and 15% drop-out during intervention/follow-up) among clusters (i.e. 0 individuals lost within one cluster, one individual lost within one cluster, two individuals lost within seven clusters, three individuals lost within 15 clusters). Since no exact formula exists for such a scenario, we performed a simulation study. We simulated 24 (number of clusters) times ten observations (subjects) from a normal distribution with mean equal to 9.66, SD equal to 14.53 and ICC=0.10. Then we randomly selected one cluster where we randomly deleted one subject, we randomly selected seven clusters where we randomly deleted two subjects each, we randomly selected 15 clusters where we randomly deleted three subjects each. We simulated 1000 samples and computed the observed statistical power. Twelve clusters per treatment arm (total number of clusters=24) with ten individuals in one cluster, nine individuals in one cluster, eight individuals in seven clusters, and seven individuals in the remaining 15 clusters (total sample size 180) achieves 91.8% power to detect a mean difference of 9.66 between the two arms in terms of change in CD-RISC 25 at 3-month follow-up (Donner A, Klar, 1996).

**Scenario 4 – In case of COVID19 restrictions**

Assuming a differential sample size per group among clusters due to COVID19 restrictions. Since no exact formula exists for such a scenario, we performed a simulation study: we simulated 18 (number of clusters) times ten observations (subjects) per each treatment arm (36 clusters, 360 observations) from two normal distributions with the same parameters as estimated in the pilot study and ICC=0.10 resulting in a treatment mean difference equal to 9.66.

Then we randomly selected 12 clusters per arm where we randomly deleted 5 subjects. We simulated 1000 samples and computed the observed statistical power. Therefore this simulated study, consisting of 12 clusters per treatment arm (24 clusters) with 5 individuals and 6 clusters per treatment arm (12 clusters) with 10 individuals (total sample size 240) achieves 99.9% power to detect a mean difference of 9.66 between the two arms in terms of change in CD-RISC 25 at 3-month follow-up (Donner A, Klar, 1996).

**Scenario 4.1:**

If a random drop-out of 10% was assumed for those 12 clusters with 10 individuals (total sample size 228), the observed power remained essentially unchanged (99.3%).

**Scenario 4.2:**

If a random drop-out of 10% and a random lack of enrollment of 10% was assumed for those 12 clusters with 10 individuals (total sample size 216), the observed power remained essentially unchanged (98.6%).​

**Reference**

Donner A, Klar N. Statistical considerations in the design and analysis of community intervention trials. J Clin Epidemiol. 1996;49(4):435–439.
