# Supplementary material for: A group resilience training program for people with multiple sclerosis: Study protocol of a multi-centre cluster-randomized controlled trial (multi-READY for MS)
Source: PLoS One. 2022 May 2;17(5):e0267245. doi: 10.1371/journal.pone.0267245 (PMC9060330; doi:10.1371/journal.pone.0267245)
Supplement: S1 File — (PDF) [file pone.0267245.s007.pdf]

Fondazione I.R.C.C.S. Istituto Neurologico Carlo Besta
Sistema Socio Sanitario
Regione Lombardia

Clinical Study Protocol

A resilience group training program for people with multiple sclerosis: multi-centre randomized controlled trial (Multi_READY for MS)

PROTOCOL NO.: TBD

Version: 1.2

ISRCTN: 67194859

Study Design: multi-centre, randomized controlled trial.

Study population: Multiple sclerosis patients

Promoting Centre and Study location: Fondazione IRCCS Istituto Neurologico Carlo Besta Via Celoria 11, 20133 Milan. Italy.

Principal Investigator: Dr. Ambra Mara Giovannetti

Operative Units:

- Unit of Neuroepidemiology (Head: Dr. Alessandra Solari)
- MS Centre, Unit of Neuroimmunology and Neuromuscular Diseases (Head of the MS Centre: Dr. Paolo Confalonieri)

Participating centres:

- San Camillo-Forlanini Hospital, Roma (Head: Dr. Carla Tortorella)
- AISM Rehabilitation Service of Genoa, Italian Multiple Sclerosis Society, Genova (Head: Dr. Giampaolo Brichetto)
- Neurology Clinic, Multiple Sclerosis Centre, University Hospital Policlinico Vittorio Emanuele, Catania, Italy (Head: Prof. Francesco Patti)
- Laboratorio di neuropsicologia, UOSD psicologia clinica e UOC neurologia, ASST Lariana (Head: Dr. Monica Grobberio)

- Centro Sclerosi Multipla, Divisione di Neurologia Generale, IRCCS Fondazione Istituto Neurologico Nazionale C. Mondino di Pavia (Head: Dr. Bergamaschi)
- Dipartimento Riabilitazione ASLUMBRIA2 (Head: Dr. Micheli)
- Centro Malattie Demielinizzanti e Laboratori di Neurologia Sperimentale, Clinica Neurologica, Università di Perugia. (Head: Dr. Di Filippo)

September 16, 2020

Tables of contents

Section	Page
1. SUMMARY	5
2. INTRODUCTION	7
2.1 From a pilot to a multi-center RCT	9
3. AIM	9
4. ENDPOINTS	9
4.1 Primary Endpoint	9
4.2 Secondary Endpoints	9
5. MEASURES TAKEN TO MINIMIZE BIAS	10
6. METHODOLOGY	11
6.1 Study design	11
6.2 Study participants and recruitment	11
6.2.1 Participant eligibility	11
6.2.2 Recruitment and trial procedures	12
6.2.3 Withdraw	12
6.2.4 Pre-study interview and informed consent (visit 0)	12
6.2.5 Assessments	12
6.3 Interventions	12
6.3.1 Italian READY for MS	13
6.3.2 Control intervention	13
6.3.3 Intervention Fidelity and supervision	13
6.4 Measures	14
6.4.1 Primary outcome measure	14
6.4.2 Secondary outcome measures	14
6.5 Data analysis	16
6.5.1 Sample size calculation	16
6.5.2 Statistics	17
7. STRATEGIES FOR LIMITING COVID-19 IMPACTS ON THE STUDY	18
7.1 Ancillary study on the COVID-19 peritraumatic distress	19
8. PANELS	20
8.1 Trial Steering Committee (TSC)	20
8.2 Independent Data and Safety Monitoring Committee (IDSMC)	20
8.3 Data Management and Analysis Committee (DMAC)	20
8.4 Clinical Psychology Expert Panel	20
9. PROJECT DURATION	20
10. EXPECTED RESULTS AND IMPACT	20
11. ETHICS AND ADMINISTRATIVE CONSIDERATIONS	20
11.1 Ethical Considerations	20
11.2 Ethics Committee approval	21
11.3 Subject Information and Informed Consent	21
11.4 Confidentiality	21
11.5 Protocol Amendments	21
12. STUDY MANAGEMENT AND MONITORING	21
12.1 Protocol deviation	21
12.2 Protocol Violation	21
12.3 Source Documents	21
12.4 Archiving of Records	21
12.5 Auditing on site	21
12.6 Use and publication of study results	21
12.7 Insurance Policy	22
13. FUNDING	22
14. REFERENCES	22

15. GLOSSARY OF ABBREVIATIONS	27
16. LIST OF APPENDICES	28

1. SUMMARY

Background: Adjusting to MS can be highly demanding, and the disease can be a consistent source of stress. Resilience is an internal resource for alleviating the adverse effects of stress and sustaining good mental health through adversity.

In recent years, an Australian team developed and tested an Acceptance and Commitment Therapy (ACT)-based group resilience-training program: the REsilience and Activities for every DaY (READY). In view of the promising preliminary data on the READY for MS resilience training intervention, we decided to apply the READY for MS in Italy and to evaluate the efficacy of the program by following the Medical Research Council (MRC) framework for developing and evaluating complex interventions.

The project is composed of two phases: 1) a pilot randomized controlled trial (RCT) with a nested qualitative study; 2) a multicenter phase III RCT, with an ancillary study on the impact of the training on the psychologists who will learn and deliver the READY for MS program.

Results from phase 1 showed that Italian READY for MS was well accepted by MS patients. Qualitative (but not quantitative) data provided evidence in favour of READY. Our findings informed methodological and intervention refinements for the multi-centre RCT (phase 2).

Aim: This study aims to evaluate the efficacy of the Italian READY for MS program in a multi-center RCT. Our primary end-point is the between-arm difference in T0 - T2 changes in CD-RISC 25 score.

Methods: This is a multi-centre RCT assessing the superiority of Italian READY for MS over relaxation in a sample of 240 people with MS. Groups will be allocated to Italian READY for MS or control intervention in a 1:1 ratio.

Study Intervention. READY for MS is an adult ACT informed group resilience training program which consists of seven weekly 2.5 hour sessions plus a 2.5 hour 'booster' session approximately five weeks after the seventh session. Content of the seven weekly sessions is as follows: an introductory module (Introduction to the READY Resilience Model), five modules focusing on each of the six ACT processes (Mindfulness, Acceptance, Cognitive Defusion, Self-as-Context, Values and Meaningful Action), and a review module (Review and Future Planning).

Control Intervention. The control condition consisted of a group relaxation program based on autogenic training. The intervention consisted of seven, 1-hour weekly group sessions, followed by a 'booster' session approximately after five weeks (S3 Appendix - Relaxation sessions). This control program matched the study intervention in number of sessions and schedule (but not in session content and length).

Trial outcomes. Patient reported outcome measures (PROMs) will be assessed immediately before, after the booster session, at three and six month follow-up. Additional process data will capture participants' attendance, homework completion, and facilitator perspectives on a weekly basis. Each session will be audio recorded and self-rated by the facilitators. Two sessions for each Italian READY for MS group will be randomly selected and independently rated by two ACT experts to assess intervention fidelity. The control group session fidelity will be assessed following the same procedures except for the use of ACT-FM and audio recording assessment that will be done by one rater.

Data analysis. Longitudinal changes will be analyzed using repeated measures hierarchical (patients nested in clusters) generalized linear mixed models, accounting for the cluster effect (using a random intercepts for clusters). All group comparisons will be carried out according to the intention-to-treat principle.

Discussion: This study will produce evidence on the efficacy of a brief, structured group intervention to promote resilience in people with MS by comparing it with an active group intervention. It is expected that, by empowering participant inner resources, Italian READY for MS can promote a personal growth that may help participants to prevent or overcome difficulties in adjustment to MS, and to live a full and rich life. The Italian READY for MS program is brief and highly structured, which ease its affordability.

Trial registration: (ISRCTN registry) TBD

Keywords: Multiple sclerosis, Resilience, Randomized controlled trial, Acceptance and Commitment Therapy, Complex intervention.

2. INTRODUCTION

Multiple sclerosis (MS) is an inflammatory, progressive demyelinating disease of the central nervous system. With a lifetime risk of 1 in 400, it is the most common cause of progressive neurological disability in young adults [1]. The most recent study on the global prevalence of MS estimated that approximately 2.3 million people worldwide have MS, with Canada, USA and some European countries, including Italy, having the highest prevalence rates [2]. The Italian MS Foundation estimated that there are more than 122,000 persons with MS (PwMS) in Italy [3]. Given that MS typically manifests in young adulthood [4], the impact of diagnosis is particularly distressing as it has the potential to significantly interfere with life goals [5], and negatively impact on work-related activities [6]. Patients' experience of MS is characterized by remissions, relapses, possible persistent disability and continuous progression. As a result, PwMS often have to deal with uncertainty about disease progression, loss of function, changes in life roles and a variety of symptoms [7]. For these reasons, adjusting to MS can be highly demanding [8], and the disease can be a consistent source of stress. In fact, PwMS have poorer quality of life (QoL) than healthy controls and people with other chronic diseases; lifetime prevalence is approximately 50% for depressive symptoms and 35% for anxiety disorders [9, 10]. In addition, evidence suggests an association between psychological stress and subsequent relapses in MS, with the occurrence of stressful life events purported to lead to a greater risk for relapses [11].

Resilience is an internal resource for alleviating the adverse effects of stress and sustaining good mental health through adversity [12]. It entails the process of negotiating, managing and adapting to significant stressors or trauma through drawing on internal (i.e. mindfulness, acceptance, cognitive flexibility and active coping), and external (i.e. social support, financial capital and community services) resources [13]. In times of adversity, people with low resilience have a higher risk of experiencing poor QoL, emotional burden and interpersonal difficulties [14]. Moreover, they can adopt health compromising behaviors and experience somatic complaints and poor physical health [15]. Prolonged stress together with poor psychosocial functioning may negatively affect physical health through different mechanisms, such as hypertension and blood pressure reactivity to stress, pro-inflammatory cytokines and the development of metabolic syndrome [16]. Given that PwMS have lower resilience than community samples and people with other chronic illnesses [17], they are particularly vulnerable to low QoL and well-being. Therefore, targeted interventions aimed at fostering resilience are crucial in helping PwMS deal with their illness-related stressors and improve their QoL.

Leppin et al's [12] meta-analysis of resilience training programs in adults showed modest but consistent benefits in improving a number of mental health outcomes. A more recent review demonstrated that resilience training promotes a range of positive psychosocial outcomes in people with chronic illnesses [18]. Resilience training programs have been shown to improve QoL, anxiety, depression, perceived stress and well-being in adults with cancer [19, 20, 21], congenital heart disease [22], diabetes [23], and neurofibromatosis [24]. Despite these promising findings, a recent narrative review of resilience training identified numerous methodological limitations. First, evidence of the benefits of resilience training remains limited; second, resilience training interventions are often not well differentiated from other forms of training; third, the effects of the training on psychological functioning are strongly influenced by the outcome measures selected and the setting of the training [25].

In recent years, an Australian team developed and tested an Acceptance and Commitment Therapy (ACT)-based group resilience-training program: the REsilience and Activities for every DaY (READY). The READY program was initially trialed in a workplace setting [26, 27] and then adapted and successfully applied to different health conditions: cancer [28] diabetes [29] and MS [30]. Findings showed READY for MS had beneficial impacts on resilience, QoL, depression, stress and protective factors (managing difficult thoughts, values and acceptance) in PwMS [30]. READY for MS is based on ACT which is a recent variant of cognitive behavior therapy. ACT is informed by the psychological flexibility framework. Psychological flexibility is defined as “the ability to contact the present moment more fully as a conscious human being, and to change or persist in behavior when doing so serves valued ends” [31]. It is fostered by six ACT processes: (1) acceptance – openness to experience, (2) cognitive defusion – observing thoughts rather than taking them literally, (3) present moment awareness – mindfulness, (4) self as context – contact with a sense of self that is continuous and provides flexible perspective taking, (5) values – freely chosen personally meaningful life directions, (6) committed action – values-guided effective action [31]. Each process has been shown to be related to better mental health, lower risk of disease, better health outcomes for those already diagnosed with illness [29, 31-34]. Psychological flexibility plays a key role in promoting resilience [35]. Furthermore, psychological flexibility processes have been shown to mediate the beneficial effects of resilience training in PwMS [30].

In view of the promising preliminary data on the READY for MS resilience training intervention and taking into consideration the limitations highlighted by Forbes and Fikretoglu [25], we decided to apply the READY for MS in Italy and to evaluate the efficacy of the program by following the Medical Research Council (MRC) framework for developing and evaluating complex interventions, which has a phased approach, from a pre-clinical research phase to a final phase in which the intervention is introduced into the health service, leading to a theory-driven intervention: a "bottom up" development which guarantee to enter a phase III trial with an appropriate theory and pilot work [36]. Further, both quantitative and qualitative methods are used and integrated within the framework, in order to better appraise the effects of the (complex) intervention as a whole and its components. For this reason, the project evaluating the Italian version of the READY for MS program is composed of two phases: 1) a pilot randomized controlled trial (RCT) with a nested qualitative study; 2) a multicenter phase III RCT, with an ancillary study on the impact of the training on the psychologists who will learn and deliver the READY for MS program.

The pilot, single blind RCT with a nested qualitative study comparing Italian READY for MS with relaxation (phase 1) was run between April 2017 and January 2018 at the Fondazione IRCCS Istituto Neurologico Carlo Besta. Four intervention groups were conducted with 39 participants (20 Italian READY for MS, 19 relaxation). Two patients assigned to Italian READY for MS withdrew before beginning the intervention due to unexpected work commitments. The Italian READY for MS program was well accepted by MS patients with varied socio-demographic and clinical characteristics, suggesting it has high utility and acceptability in an Italian clinical setting. There was an improvement of the primary and in most of the secondary outcome measures over time, which did not differ statistically between the Italian READY for MS and group relaxation. Qualitative data indicated that Italian READY for MS was viewed as superior to relaxation by participants assigned to relaxation who, at the end of the trial, received Italian READY for MS. This finding converges with four non-significant statistical trends supporting the efficacy of Italian

READY for MS. Consistent with the ACT psychological flexibility framework, the qualitative data indicated that participants' perceived improvements in resilience and QoL were due to the acquisition of skills related to the six core ACT processes. Findings informed methodological and intervention refinements for the multi-centre RCT evaluation of the Italian READY for MS program (phase 2).

2.1 From a pilot to a multi-center RCT

Data from the Pilot RCT were discussed in a dedicated meeting with an International Panel. The meeting focused on the design of the next multi-centre READY for MS RCT. It was a structured discussion, using a "PICO" (Population, Intervention, Comparator, Outcomes) format [37]. The panel reached the following conclusions:

Population. The eligibility criteria of the pilot RCT were deemed adequate by the panel.

Intervention. The panel agreed on confirming the program in its current format.

Comparator. Participants will be allocated to Italian READY for MS or control group (relaxation) in a 1:1 ratio. Control intervention facilitators should not have training in mindfulness or ACT, in order to prevent contamination. The experimental and comparator interventions will be delivered by different people.

Outcome measures. Following Chmitorz et al. suggestion [38] and based on the trend differences observed in the pilot RCT, the Connor-Davidson Resilience Scale 25 (CD-RISC 25) [39] was chosen as primary outcome.

The sample size presented in this study protocol was calculated on pilot RCT data in terms of change in CD-RISC 25 at 3-month follow-up, and using a conservative approach (assuming an Intraclass Correlation (ICC) equal to 0.10, instead of .07; power higher than .91, instead of .80; assuming a drop-out rate of 25%.

3. AIM

This study aims to evaluate the efficacy of the Italian READY for MS program in a multi-center RCT. We hypothesize that participants in the Italian READY for MS would show higher improvements on the primary outcome of resilience (CD-RISC 25 [39]) and on the secondary outcomes of mood (The Hospital Anxiety and Depression Scale, HADS [40]; The Positive and Negative Affect Schedule, PANAS [41]), health related quality of life (54-items MS Quality of Life inventory, MSQOL-54 [42]; the European Quality of life Five Dimensions, EQ-5D-3L [43]), well-being (The short form of the Mental Health Continuum, MHC-SF [44]), and psychological flexibility (The Comprehensive assessment Acceptance and Commitment Therapy processes, CompACT [45]), compared to the control group (relaxation).

4. ENDPOINTS

4.1 Primary Endpoint

Our primary end-point is the between-arm difference in T0 - T2 changes in CD-RISC 25 score.

4.2 Secondary Endpoints

- Differences between changes in CD-RISC 25 scores at post intervention (T1) and 6-month follow-up (T3) between Italian READY for MS and relaxation groups.

- Differences between changes in HADS, PANAS, MSQOL-54 (mental health component, MHC; physical health component, PHC), EQ-5D-3L, MHC-SF, CompACT scores at different time-points between Italian READY for MS and relaxation groups.

5. MEASURES TAKEN TO MINIMIZE BIAS

The study follows the CONSORT guidance for RCTs on social and psychological interventions (CONSORT-SPI 2018) [46]. This includes the devise of the study protocol following the SPIRIT guidance [47], protocol's publication, and the trial public registration (ISRCTN registry).

Blinding of patients is not possible due to the type of the study intervention. The statistical analyses will be performed by study personnel blind to the participants' randomization to either the intervention group or the control group and who are not involved in providing treatment. All study personnel will be trained to conform to Good Clinical Practice (GCP) regulation. Electronic version of the study PROMs will be used to ensure the high quality of the entered data. The Coordinating Unit will monitor adherence to the study protocol and overall study quality. Finally, an Independent Data and Safety Monitoring Committee (IDSMC) will oversee the study procedures, recruitment, and data flow. The Clinical Psychology Expert Panel will monitor the intervention fidelity audio recording and rating Italian READY for MS and Relaxation sessions.

The following meetings are scheduled:

1. *Investigators meeting.* It will be run via teleconference. All the centres PIs, facilitators (both Italian READY for MS and control group) and Trial Steering Committee (TSC) members will be invited. The aim of this meeting is to provide clear information on the study procedures.
2. *Facilitators meetings.* Facilitators will receive detailed information only on the group intervention (Italian READY for MS or relaxation) they will run. For this reason, facilitator meetings will be run separately for Italian READY for MS and relaxation facilitators.
    a. *Relaxation facilitator meetings.*
        i. A teleconference dedicated to the presentation of the relaxation program will be organized before the beginning of the intervention.
        ii. A second meeting will be run after the 3rd session in order to monitor possible difficulties, top up facilitators motivation and provide a safe place for peer-discussion on the application of the relaxation program.
    b. READY for MS facilitator meetings.
        i. A two-day residential training will be offered to READY for MS facilitators before the beginning of the intervention. The meeting aims to provide a safe place for experiential exercise and peer-discussion on the application of the Italian READY for MS program. The meeting will be run by AMG and NP.
        ii. A second meeting will be run after the 3rd session in order to monitor possible difficulties, top up facilitators motivation and provide a safe place for peer-discussion on the application of the Italian READY for MS program.

    Study PI and the other member of the Clinical Psychology Expert Panel will be also available for inquiries about the application of the two programs.

6. METHODOLOGY

6.1 Study design

This is a multi-centre RCT (S1 Appendix - Multi_READY for MS Flowchart) assessing the superiority of Italian READY for MS over relaxation. Patient reported outcome measures (PROMs) will be assessed immediately before (baseline visit, T0), after the booster session (T1, 12 weeks after baseline visit), at three (T2, 24 weeks after baseline visit), and six month follow-up (T2, 36 weeks after baseline visit). Additional process data will capture participants' attendance, homework completion, and facilitator perspectives on a weekly basis. Each session (READY for MS and relaxation) will be audio recorded and self-rated by the facilitators. Two sessions for each facilitators will be randomly selected and independently rated by two ACT experts (AMG, GP) to assess intervention fidelity. The control group session fidelity will be assessed following the same procedures except for the use of ACT-FM and audio recording assessment that will be done by one rater (study PI).

After obtaining the Human Research Ethics Committee approval of each participating centre, the study will be performed at the MS/rehabilitation centre of the following centres: Fondazione IRCCS Istituto Neurologico Carlo Besta, Milano; San Camillo-Forlanini Hospital, Roma; AISM Rehabilitation Service of Genoa, Italian Multiple Sclerosis Society, Genova; Neurology Clinic, Multiple Sclerosis Centre, University Hospital Policlinico Vittorio Emanuele, Catania; Laboratorio di neuropsicologia, UOSD psicologia clinica e UOC neurologia, ASST Lariana, Como; Centro Sclerosi Multipla, Divisione di Neurologia Generale, IRCCS Fondazione Istituto Neurologico Nazionale C. Mondino, Pavia; Dipartimento Riabilitazione ASLUMBRIA2; Centro Malattie Demielinizzanti e Laboratori di Neurologia Sperimentale, Clinica Neurologica, Università di Perugia.

6.2 Study participants and recruitment

6.2.1 Participant eligibility

Inclusion criteria

- Diagnosis of MS [48]
- Age ≥18 years
- Written informed consent
- Resilience score <83
- Able to attend group sessions, and fluent Italian speaker

Exclusion criteria

- Severe cognitive compromise (Mini Mental State Examination <19)
- Psychosis or other serious psychiatric conditions
- Psychotherapy in the preceding six months
- Prior formal training in mindfulness methods or current meditation practice
- Severe suicidality, including ideation, plan and intent
- One or more relapses in the previous month
- Corticosteroid treatment during the previous month
- Other serious medical disorders in addition to MS
- Current pregnancy
- MS diagnosis for less than three months

6.2.2 Recruitment and trial procedures

A flyer, which includes a general overview of the study and contact details, will be sent via e-mail to the patients of the participating MS Centres by the MS Centre team. People who show interest in participating in the study will be contacted by the study coordinator or centre Principal Investigator (PI). Subsequently, one trained clinical psychologist (Italian READY for MS facilitator) makes an appointment with those patients who met the inclusion criteria and agreed to participate in the study, and checks all eligibility criteria. She/he sends an e-mail to the participant with the link to the website containing the set of questionnaires. The total assessment will last about 55 minutes in each time-points (additional 20 minutes at T1 and T2 for filling in the satisfaction questionnaire). Each centre will collect information on the number of people approached, screened, and eligible prior to random assignment, with reasons for non-enrolment. Participants will be then assigned to the Italian READY for MS or control condition. Randomization will be provided by an independent randomization unit, using computer-based cluster randomization with minimization [49] (2 factors: Centre and CDRISC score < 50 and ≥ 50). Groups will be allocated to Italian READY for MS or control intervention in a 1:1 ratio. Confirmation e-mails will be sent to the study coordinator and centre PI. The interventions will start within two weeks of the baseline assessment.

6.2.3 Withdraw

Participants will be free to withdraw from the study at any time, without giving reasons and with no risk of prejudicing future care. Study personnel will make every effort to obtain, and record, information about the drop out reasons.

6.2.4 Pre-study interview and informed consent (visit 0)

During the pre-study evaluation each potential participant receives full and adequate verbal and written information about the nature and purpose of the study. A written, signed informed consent is obtained, according to the Declaration of Helsinki and to the GCP Guidelines of the EU. The informed consent form will be kept on file by the study personnel and will be available for inspection by regulatory authorities or authorized persons.

6.2.5 Assessments

PROMs will be assessed immediately before (baseline visit, T0), after the booster session (T1, 12 weeks after baseline visit), at three (T2, 24 weeks after baseline visit), and six month follow-up (T2, 36 weeks after baseline visit) via web survey. The satisfaction questionnaire will be administered at T1 and T2. Participants will receive an e-mail with the link to the website containing the set of questionnaires.

6.3 Interventions

Each group will be composed of 8-10 participants, a total of 8 groups will be performed (four Italian READY for MS and four relaxation); within each arm, the groups will be homogeneous assembled so that PwMS will be as much homogeneous as possible in terms of CD-RISC 25 scores. The Italian READY for MS groups will be run by a psychologist of the "Rete Psicologi AISM" who participated and successfully completed "The ACT and be READY for MS training program". To

limit possible involuntary contaminations, the control group will be run by a psychologist not involved in "The ACT and be READY for MS training program" and with no expertise in ACT or mindfulness interventions. Each group will be run in a dedicated room of the Hospital/Institute of each participating MS Centre. Participants will be asked not to disseminate information or materials of the program in which they are allocated. In addition, READY for MS and relaxation groups will be scheduled in order to prevent contact between participants of the two groups. Facilitators will be asked not to disseminate information or materials with the colleagues who are running the other intervention.

6.3.1 Italian READY for MS

READY for MS is an adult ACT informed group resilience training program which consists of seven weekly 2.5 hour sessions plus a 2.5 hour 'booster' session approximately five weeks after the seventh session (S2 Appendix - READY for MS sessions). Content of the seven weekly sessions is as follows: an introductory module (Introduction to the READY Resilience Model), five modules focusing on each of the six ACT processes (Mindfulness, Acceptance, Cognitive Defusion, Self-as-Context, Values and Meaningful Action), and a review module (Review and Future Planning). The booster session provides a review of the program content. The program has a facilitator manual, participant workbook, and audio recordings of mindfulness exercises. Throughout the program, participants are encouraged to share their progress and experience of applying the READY strategies and techniques. It incorporates a blend of psychoeducation and experiential exercises, combined with readings and homework exercises that participants are encouraged to practice between sessions [30].

6.3.2 Control intervention

The control condition consisted of a group relaxation program based on autogenic training [50]. The intervention consisted of seven, 1-hour weekly group sessions, followed by a 'booster' session approximately after five weeks (S3 Appendix - Relaxation sessions). This control program matched the study intervention in number of sessions and schedule (but not in session content and length) in order to control for the non-specific effects of Italian READY for MS. The program had a facilitator manual, participant workbook, and audio recordings of relaxation exercises.

6.3.3 Intervention Fidelity and supervision

Each session (Italian READY for MS and relaxation) will be audio recorded. The file will be sent via e-mail to the study PI immediately after each session.

The procedure to monitor fidelity include the following actions.

After each session, facilitators fill in a purpose-build checklist (S4 Appendix – READY for MS Session Fidelity Checklist; S5 Appendix – Relaxation Session Fidelity Checklist) and the "The Acceptance and Commitment Therapy Fidelity Measure (ACT-FM)" [51] (only Italian READY for MS facilitator), and send it to study PI.

After each session, the study PI will read the checklists, see if there are self-reported discrepancies with the manual and/or inconsistencies with ACT principles (only for Italian READY for MS facilitators). In case of self-reported discrepancy (or inconsistency), the study PI will contact the facilitator and discuss about the session. She will be available for supervision anytime.

For the READY for MS group, two independent raters (AMG and GP) will listen to the audio of the session and evaluate the intervention fidelity in two sessions that will be randomly selected for each facilitators: one at the beginning of the program (session 2 - Mindfulness or Session 3 - Acceptance) and one from the second part of the program (session 4 - 7). Intervention fidelity will be assessed by filling in the pertinent purpose-build checklist and the ACT-FM" [51]. If the raters evaluate the quality of the session as low, the study PI will contact the facilitator to critically discuss about the session and the two raters will evaluate the quality of her/his conduction of the next session.
We will consider low fidelity if at least one of the following criteria will be satisfied:

1. There is a discrepancy with the content manual in more than one section of the READY for MS Session Fidelity Checklist.
2. Therapists must get a score at the ACT-FM of less than 5 out of 9 for the Stance Consistent section (items 1 -4) and a score of less than 11 out of 27 for the other sections combined [this criteria were defined after discussion with the ACT-FM Authors].

The control group session fidelity will be assessed following the same procedures except for the use of ACT-FM and audio recording assessment will be done by one rater (study PI)

6.4 Measures

We will use the Italian versions of the HADS [52], PANAS [53], MSQOL-54 [54], EQ-5D-3L [55], MHC-SF [56]. For the CD-RISC 25 we will use the unpublished Italian version [courtesy of Davidson]. The CompACT was translated into Italian for the previous pilot RCT [paper in preparation]. These PROMs will be administered in the order presented in the following section.

6.4.1 Primary Outcome Measure

Resilience. The CD-RISC 25 is used to assess psychological resilience. It is composed of 25 items, each rated on a 5-point scale (0-4), with higher scores reflecting greater resilience. The scale demonstrated good psychometric properties [39].

6.4.2 Secondary Outcomes

Mood

- The HADS is a well-validated measure that consists of two seven-item subscales to assess anxiety and depressive levels. Higher scores indicate higher levels of depressive or anxiety symptoms [40]. Unlike many similar measures, the HADS excludes somatic symptoms of anxiety and depression, which may overlap with physical illness [40].
- PANAS consists of two 10-item mood scales and it is a self-report measure of positive and negative affect. Respondents are asked to rate the extent to which they have experienced each particular emotion within a specified time period, with reference to a 5-point scale. The scale point are: 1 'very slightly or not at all', 2 'a little', 3 'moderately', 4 'quite a bit' and 5 'very much'. The PANAS resulted to be a reliable and valid measure of the constructs it was intended to assess [41]

Health related quality of life.

- The MSQOL-54 is a health-related QoL measure that comprises the generic Short-Form 36-item (SF-36), plus 18 MS-specific items [42]. The 54 items are organized into 12 multi-item and two single item subscales. As for the SF-36, two composite scores (Physical Health Composite, PHC, and Mental Health Composite, MHC) are derived by combining scores of the relevant subscales. The MSQOL-54 has well documented validity in terms of content, constructs, reliability [42], discrimination and responsiveness [57]. To limit multiple comparisons, we primarily assessed changes in the MHC.
- The EQ-5D-3L is a preference-based health-related QoL measure with one question for each of the five dimensions that include mobility, self-care, usual activities, pain/discomfort, and anxiety/depression. It also includes a Visual Analog Scale (VAS), to report perceived health status with a grade ranging from 0 (the worst possible health status) to 100 (the best possible health status) [43].

Well-being

MHC-SF measures positive mental health and comprises 14 items, representing various feelings of well-being. Respondents rate the frequency of every feeling in the past month on a 6-point Likert scale (never, once or twice a month, about once a week, two or three times a week, almost every day, every day). The MHC-SF has shown good psychometric properties [44].

General measure of ACT processes

The 23-item CompACT has three subscales: openness to experience (OE), behavioral awareness (BA), valued action (VA). A total score is calculated by summing the three subscale scores. Items are rated on a 7-point Likert scale. The full-scale CompACT total score ranges from 0-138, with higher scores indicating greater psychological flexibility. The CompACT demonstrated good internal consistency, and converged and diverged in theory-consistent ways with other measured variables: higher levels of psychological inflexibility were associated with higher distress and lower health and well-being [45].

Clinical information and measures

The following information will be also provided by the PwMS neurologist at T0: EDSS score [58], MS course (relapsing remitting, primary progressive, secondary progressive), presence/type of co-pathologies, and ongoing treatment. The neurologist will update occurrence of new relapses at each time-point.

Satisfaction with the intervention

Four purpose-build questionnaires (two for Italian READY for MS; two for relaxation) explore the satisfaction with the Italian READY for MS program or relaxation.

- “Satisfaction with the Italian READY for MS – T1” questionnaire is composed of four sections: 1) Usefulness of the Italian READY for MS program in promoting the six protective factors of resilience (5-item); 2) Overall evaluation of the Italian READY for MS program (6-item); 3) Satisfaction with the READY Personal Plan (5-Item); 4) Participants personal experience with the Italian READY for MS (8-open questions on their experience).

- “Satisfaction with the Italian READY for MS – T2” questionnaire is composed of three sections: 1) Usefulness of the Italian READY for MS program in promoting the six protective factors of resilience (5-item). 2) Overall evaluation of the Italian READY for MS program (6-item); 3) Participants personal experience with the Italian READY for MS (10-open questions on their experience).
- “Satisfaction with the relaxation program – T1” questionnaire is composed of two sections: 1) Overall evaluation of the relaxation program (5-item); 2) Satisfaction with the relaxation Personal Plan (5-Item).
- “Satisfaction with the relaxation program – T2” questionnaire is composed of 5-item and 2 open ended questions on the overall evaluation of the relaxation program.

Adherence to the intervention

The facilitator collect information on participant’s retention and ask the participants to rate the level of commitment with the homework activities (READY Personal Plan; Relaxation Personal Plan), after each session.

Intervention fidelity

- The Session Fidelity Checklist is a purpose-build checklist that reflects the sequences of session components as reported in the Facilitator Manual. It also includes a section for clinical notes. Two versions are available, one for the Italian READY for MS (S4 Appendix – READY for MS Session Fidelity Checklist), and on for the relaxation program (S5 Appendix – Relaxation Session Fidelity Checklist).
- “The Acceptance and Commitment Therapy Fidelity Measure (ACT-FM)” is 25-item checklist on a 4-point Likert scale that evaluates the adherence with the ACT therapeutic style. It is composed of two total scores (Total ACT Consistency Score (0-36) = Total ACT Inconsistency Score) and eight subscales that range from 0 to 9, namely: 1) ACT Consistent Therapist Stance; 2) ACT Inconsistent Therapist Stance; 3) ACT Consistent Open Response Style; 4) ACT Inconsistent Open Response Style; 5) ACT Consistent Aware Response Style; 6) ACT Inconsistent Aware Response Style; 7) ACT Consistent Engaged Response Style; 8) ACT Inconsistent Engaged Response Style [51].

6.5 Data analysis

6.5.1 Sample size calculation

The sample size calculation was based on the results from the pilot study [paper under review], where we found a mean change in the CD-RISC 25 at 3-month follow-up in the READY for MS arm equal to 15.61 (standard deviation, SD: 13.63, n=18) vs. a mean change in the relaxation arm equal to 5.95 (SD: 14.11, n=19). Moreover, we estimated an intra-cluster correlation coefficient (ICC) equal to 0.08.

A sample size of 12 clusters per treatment arm (total number of clusters=24) with 10 individuals per cluster (total sample size 240) achieves 94.6% power to detect a mean difference of 9.66 between the two arms in terms of change in CD-RISC 25 at 3-month follow-up [59]. The SD of change in the CD-RISC 25 at 3-month is assumed equal to 14.53. The ICC is assumed, conservatively, equal to 0.10. The type I error (alpha) is assumed to 0.05.

Starting from the previous scenario (i.e. total sample size 240, 24 clusters, 10 subjects per cluster), we computed the study power assuming three scenarios of drop-out.

Scenario 1
Assuming a drop-out of 20% uniformly distributed among clusters (i.e. two individuals lost within each cluster): 12 clusters per treatment arm (total number of clusters=24) with eight individuals per cluster (total sample size 192) achieves 92.1% power to detect a mean difference of 9.66 between the two arms in terms of change in CD-RISC 25 at 3-month follow-up [59]. The SD of change in the CD-RISC 25 at 3-month is assumed equal to 14.53. The ICC is assumed, conservatively, equal to 0.10. The type I error (alpha) is assumed to 0.05.

Scenario 2
We assumed a variables drop-out of 20% (e.g. 5% failure to reach the enrollment target of 10 participants per group and 15% drop-out during intervention/follow-up) among clusters (i.e. 0 individuals lost within two clusters, one individual lost within five clusters, two individuals lost within eight clusters, three individuals lost within nine clusters). Since no exact formula exists for such a scenario, we performed a simulation study. We simulated 24 (number of clusters) times ten observations (subjects) from a normal distribution with mean equal to 9.66, SD equal to 14.53 and ICC=0.10. Then we randomly selected five clusters where we deleted randomly one subject, we randomly selected eight clusters where we deleted randomly two subjects each, we randomly selected nine clusters where we deleted randomly three subjects each. We simulated 1000 samples and computed the observed statistical power. Twelve clusters per treatment arm (total number of clusters=24) with 10 individuals in two clusters, nine individuals in five clusters, eight individuals in eight clusters, and seven individuals in the remaining nine clusters (total sample size 192) achieves 92.0% power to detect a mean difference of 9.66 between the two arms in terms of change in CD-RISC 25 at 3-month follow-up [59].

Scenario 3
We assumed a variables drop-out of 25% (e.g. 10% failure to reach the enrollment target of 10 participants per group and 15% drop-out during intervention/follow-up) among clusters (i.e. 0 individuals lost within one clusters, one individual lost within one clusters, two individuals lost within seven clusters, three individuals lost within 15 clusters). Since no exact formula exists for such a scenario, we performed a simulation study. We simulated 24 (number of clusters) times ten observations from a normal distribution with mean equal to 9.66, SD equal to 14.53 and ICC=0.10. Then we randomly selected one cluster where we deleted randomly one subject, we randomly selected seven clusters where we deleted randomly two subjects each, we randomly selected 15 clusters where we deleted randomly three subjects each. We simulated 1000 samples and computed the observed statistical power. Twelve clusters per treatment arm (total number of clusters=24) with 10 individuals in one cluster, nine individuals in one cluster, eight individuals in seven clusters, and seven individuals in the remaining 15 clusters (total sample size 180) achieves 91.8% power to detect a mean difference of 9.66 between the two arms in terms of change in CD-RISC 25 at 3-month follow-up [59].

6.5.2 Statistics

Descriptive statistics will be calculated for general and clinical variables. Specifically, continuous variables will be summarised by their mean and SD, or median and interquartile range; categorical variables will be summarised as numbers and percentages.

The normality assumption will be tested with the Shapiro-Wilk test. Between-group comparisons will be carried out using either the two-sided unpaired t-test or the Wilcoxon two sided two-sample test for continuous variables depending on data distribution, and the chi-squared test for categorical variables. Correlations will be computed using Spearman's or Pearson's coefficients depending on data distribution.

Our primary end-point is the between-arm difference in T0 - T2 changes in CD-RISC 25 score.

Longitudinal changes will be analyzed using repeated measures hierarchical (patients nested in clusters) generalized linear mixed models, accounting for the cluster effect (using a random intercepts for clusters). To mitigate the potential risk of inflating the type I error in presence of a small number of clusters, we will use the Kenward-Roger degrees of freedom correction, which does not rely on the assumption of fixed cluster sizes [60].

All group comparisons will be carried out according to the intention-to-treat principle, i.e. participants will be analysed in the arm (READY for MS or relaxation) to which they were randomized. In addition, we will: carry out a per protocol analysis; assess the sensitivity of the results to excluding patients who missed three or more READY for MS group sessions. No interim analysis has been planned.

Inter-rater reliability will be assessed with the weighted kappa statistic for ordinal data, and the ICC for continuous data [61-63]; 95% confidence intervals (CIs) will be determined using the bootstrap method (1000 replications).

Missing data

Sensitivity analyses will be performed if missing data will be present. The above mentioned statistical analyses will be re-run imputing missing data according to each of the following scenarios: 1) Baseline observation carried forward; 2) Last obseration carried forward (LOCF); 3) Multiple imputation of multilevel data [64].

7. STRATEGIES FOR LIMITING COVID-19 IMPACTS ON THE STUDY

Conducting a study that involves face-to-face group interventions during the COVID-19 pandemic exposes to the risk of having to abort interventions due to the emergence of local COVID-19 infection 'hot-spots'. The TSC carefully considered three options. First, pausing the study until the pandemic situation is under control. Due to the uncertainties of predictions about when the pandemic will be more manageable, this option was excluded. Second, delivering the interventions online. This option was also excluded because it would violate the MRC framework guidelines by nullifying the pilot RCT that, as purposed, provided data that informed the design of the present multi-centre RCT. Third, retaining the group delivery of both interventions with an option for teleconference delivery in the case of COVID-19 infection threats. This option was accepted by the TSC and involved the following set of actions.

- The study PI will have weekly contact with the participating centres in order to monitor changes in the local spread of COVID-19 and to take prompt infection control actions as necessary in accordance with guidelines from the Italian Government and local health authorities.
- In the case of one or more participating centres not being able to start the study up to four months after the commencement of enrolling participants in other centres, the TSC will propose the active centres to run the remaining groups in their place (competitive enrollment)
- If face-to-face group meetings are interrupted because of COVID-19 related-issues (e.g., local lockdown), all affected participants will be invited to complete their intervention via teleconferencing and the assessments completed as scheduled.
- Relevant COVID-19 information will be collected (i.e., COVID-19 infected; family member infected; COVID-19 impacts on employment; risk perceptions of COVID-19 infection because of MS; perceived additional burden of MS treatment/rehabilitation because of COVID-19).
- During data analysis a sensitivity analysis will be undertaken to explore any effects of the teleconference intervention delivery relative to the face-to-face delivery.

In addition to the strategies reported above, the Trial Steering Committee decided to run an ancillary study on the impact of the two interventions on the COVID-19 peritraumatic distress.

7.1 Ancillary study

7.1.1 Aim

The aim of this ancillary study is to verify that participants in the Italian READY for MS would show higher improvements on the COVID-19 Peritraumatic Distress Index (CPDI) [65], compared to the control group (relaxation).

7.1.2 Endpoint

The primary endpoint will be the between-arm difference in T0 - T2 changes in CPDI score.

7.1.3 Methods

All the participants in the multi-centre RCT will be offered to participate in this ancillary study and asked to fill in the Italian version of the COVID-19 Peritraumatic Distress Index (CPDI) at baseline and 3-month follow-up (primary endpoint) [65].

Measure

The CPDI is composed of 24 items rated on a 5-point scale (0-4), measuring avoidance, compulsive behaviour, physical symptoms and loss of social functioning in the past week. A total score (0 – 100) is calculated by summing all item scores plus "4", with higher scores indicating higher COVID-19 peritraumatic distress. A score below 28 indicates no distress, between 28 and 51 mild to moderate distress, and above 51 severe distress [66].

Analysis

Longitudinal changes will be analyzed using repeated measures hierarchical (patients nested in clusters) generalized linear mixed models, accounting for the cluster effect (using a random intercepts for clusters).

8. PANELS

8.1 Trial Steering Committee (TSC)

The TSC is the executive body for the trial. Members are Dr. AM Giovannetti, Dr. M Messmer Uccelli, Prof. K Pakenham, Prof. G Presti, and Dr. A Solari.

8.2 Independent Data and Safety Monitoring Committee (IDSMC)

The IDSMC has been established to: (1) oversee the progress of the trial, and ensure that it is conducted, recorded, and reported in accordance with the protocol, GCP, and the applicable regulatory requirement(s); (2) monitor and supervise the progress of the trial, and the safety data. The IDSMC members will be a methodologist, a psychologist and an expert in complex intervention. Members are Prof. S Gold, Prof. M Bassi, and Prof. MP Sormani.

The IDSMC is scheduled to meet (teleconference) at the end of the enrollment (by the end of October 2020 and by the end of February 2021), at the end of the intervention (by the end of January 2021, and by the end of April 2021), and at the end of the follow-up (June 2021, and October 2021), and depending on the needs of the trial. One week prior to each teleconference, the trial PI will send each IDSMC member a report with the following data (overall and by site, plus other information if needed): recruitment rates, reasons for exclusion, reason for drop out. The IDSMC should report in writing to the TSC, usually within 3 weeks after the teleconference.

8.3 Data Management and Analysis Committee (DMAC)

The DMAC is responsible for data entry, quality assurance, and the statistical analyses.

The members are Dr. M Copetti (trial statistician), Dr. A Giordano, Dr. AM Giovannetti, and Dr. A Solari.

8.4 Clinical Psychology Expert Panel

An expert panel will oversee the program implementation and very its fidelity. The members are Dr. AM Giovannetti, Prof. K Pakenham, Dr. J Pöttgen and Prof. G Presti.

9. PROJECT DURATION

The project lasts 22 months (S6 Appendix – Multi_READY for MS GANTT). Possible delays due to COVID-19 pandemic will be discussed with the funding source (FISM)

10. EXPECTED RESULTS AND IMPACT

This study will produce evidence on the efficacy of a brief, structured group intervention to promote resilience in people with MS by comparing it with an active group intervention. It is expected that, by empowering participant inner resources, Italian READY for MS can promote a personal growth that may help participants to prevent or overcome difficulties in adjustment to MS, and to live a full and rich life. The Italian READY for MS program is brief and highly structured, which ease its affordability.

11. ETHICS AND ADMINISTRATIVE CONSIDERATIONS

11.1 Ethical Considerations

This clinical study was designed and shall be implemented and reported in accordance with the ICH Harmonized Guidelines for GCP, with applicable local regulations, and with the ethical principles laid down in the Declaration of Helsinki.

11.2 Ethics Committee Approval

The protocol, Subject Information Sheet, Informed Consent Form and any advertisement for the recruitment of subjects must be reviewed and approved by an appropriately constituted Ethics Committee (EC), as required in chapter 3 of the ICH E6 Guideline. Written EC approval must be obtained by the Sponsor prior to shipment of study agent or subject enrolment.

11.3 Subject Information and Informed Consent

Eligible subjects may only be included in the study after providing written (witnessed, where required by law or regulation), EC-approved informed consent, or, if incapable of doing so, after such consent has been provided by a legally acceptable representative of the subject. In cases where the subject’s representative gives consent, the subject should be informed about the study to the extent possible given his/her understanding. If the subject is capable of doing so, he/she should indicate assent by personally signing and dating the written informed consent document or a separate assent form. Informed consent must be obtained before conducting any study-specific procedures (i.e. all of the procedures described in the protocol). The process of obtaining informed consent should be documented in the subject source documents. No study procedure can be performed before the written informed consent has been provided.

11.4 Confidentiality

The investigator must ensure participant anonymity. On database and other documents, PwMS must not be identified by name but by patient number and initials. The investigator must keep a separate log of PwMS codes, dyad names and addresses, and signed informed consent forms, all of which must be kept strictly confidential.

Patient medical information obtained by this study is confidential and may only be disclosed to third parties as permitted by the Informed Consent Form (or separate authorization for use and disclosure of personal health information) signed by the patient, unless permitted or required by law. Medical information may be given to a patient‘s personal physician or other appropriate medical personnel responsible for the patient‘s welfare, for treatment purposes. Data generated by this study must be available for inspection upon request by representatives of the national and local health authorities, monitors, representatives, and collaborators, and the IRB/EC for each study site, as appropriate.

11.5 Protocol Amendments

Any protocol amendments will be prepared by the PI. Protocol amendments will be submitted to the EC and to regulatory authorities in accordance with local regulatory requirements. Approval must be obtained from the EC and regulatory authorities (as locally required) before implementation of any changes, except for changes necessary to eliminate an immediate hazard to patients or changes that involve logistical or administrative aspects only (e.g. change in monitor or contact information).

12. STUDY MANAGEMENT AND MONITORING

12.1 Protocol deviation

Accidental or unintentional changes to, or non-compliance with the research protocol that does not increase risk or decrease benefit or; does not have a significant effect on the subject's rights, safety or welfare; and/or on the integrity of the data. Deviations may result from the action of the subject, researcher, or research staff. A deviation may be due to the research subject's non-adherence, or an unintentional change to or non-compliance with the research protocol on the part of a researcher. Examples of a deviation include: A rescheduled study visit; failure to collect an ancillary self-report questionnaire; subject's refusal to complete scheduled research activities.

12.2 Protocol Violation

Accidental or unintentional change to or non-compliance with the Institute Research Board approved protocol without prior sponsor and Institute Research Board approval. Violations generally increase risk or decrease benefit, affects the subject's rights, safety, or welfare, or the integrity of the data. Examples of protocol violations: Failure to obtain valid informed consent (e.g., obtained informed consent on a non-date stamped form); accidental changes of the intervention program (Italian READY for MS or relaxation); not following inclusion/exclusion criteria.

12.3 Source Documents

Source Documents are defined as original documents, data and records. These may include hospital records, medical records / outpatient data / information laboratory, data recorded from automated instruments, etc. Investigators should conserve all the source documents as required in the study protocol for at least two years after the end of the study.

12.4 Archiving of Records

The investigator is responsible for recording and storing the essential documents of the study, according to what / and for the time required by law and by GCP. The Investigator must maintain adequate and accurate records to enable the conduct of the study to be fully documented, including but not limited to the protocol, protocol amendments, Informed Consent Forms, and documentation of EC and governmental approval. In addition, at the end of the study, the Investigator will receive the patient data, which includes an audit trail containing a complete record of all changes to data.

12.5 Auditing on Site

In the event that the investigator will be contacted by the Competent Authority in relation to this study, he or she will be required to immediately notify the Sponsor. The investigator must be available to respond to requests and queries by inspectors during the audit process. The investigator must provide the Sponsor copies of all correspondence that may affect the revision of the current study.

12.6 Use and Publication of Study Results

The results of the study may be presented during scientific symposia or published in a scientific journal only after review and written approval by the involved parties in full respect of the privacy of the participating subjects.

12.7 Insurance Policy

The Fondazione IRCCS Istituto Neurologico Carlo Besta has an adequate insurance policy to cover possible damages emerging from this study.

13. FUNDING

Supported by FISM - Fondazione Italiana Sclerosi Multipla – cod. 2016/B/3 and financed or co-financed with the '5 per mille' public funding. The funding source had no role in study design, data collection, data analysis, data interpretation or report writing.

14. REFERENCES

1. Compston A, Coles A. Multiple sclerosis. Lancet. 2002;359: 1221–1231.
2. Browne P, Chandraratna D, Angood C, Tremlett H, Baker C, Taylor BV, et al. Atlas of Multiple Sclerosis 2013: A growing global problem with widespread inequity. Neurology. 2014;83(11):1022-4. doi:10.1212/WNL.0000000000000768
3. Associazione Italiana Sclerosi Multipla Onlus (2019). Barometro della Sclerosi Multipla 2019. Available from https://www.aism.it/sites/default/files/Barometro_della_SM_2019estratto.pdf
4. Dupont S. Multiple sclerosis. In: Ayers S, Baum A, McManus C, Newman S, Wallston K, Weinman J, Frontmatterl RW, editors. Cambridge handbook of psychology, health and medicine; London: Cambridge Univ Press; 1997. pp. 5385–5440.
5. Giordano A, Granella F, Lugaresi A, Martinelli V, Trojano M, Confalonieri P, et al. Anxiety and depression in multiple sclerosis patients around diagnosis. J Neurol Sci. 2011;307(1-2): 86-91. doi: 10.1016/j.jns.2011.05.008
6. Schiavolin S, Leonardi M, Giovannetti AM, Antozzi C, Brambilla L, Confalonieri P, et al. Factors related to difficulties with employment in patients with multiple sclerosis: a review of 2002–2011 literature. Int J Rehabil Res. 2013;36: 105–111. doi: 10.1097/MRR.0b013e32835c79ea
7. Dennison L, Moss-Morris R, Chalder T. A review of psychological correlates of adjustment in patients with multiple sclerosis. Clin Psychol Rev. 2009;29(2): 141-53. doi:10.1016/j.cpr.2008.12.001
8. Aikens JE, Fischer JS, Namey M, Rudick RA. A replicated prospective investigation of life stress, coping, and depressive symptoms in multiple sclerosis. J Behav Med. 1997;20: 433-45.
9. Siegert RJ, Abernethy DA. Depression in multiple sclerosis: a review. J Neurol Neurosurg Psychiatry 2005;76: 469-75.
10. Korostil M, Feinstein A. Anxiety disorders and their clinical correlates in multiple sclerosis patients. Mult Scler J. 2007:13; 67-72.
11. Mohr DC, Hart SL, Julian L, Cox D, Pelletier D. Association between stressful life events and exacerbation in multiple sclerosis: a meta-analysis. BMJ. 2004;328(7442): 731-735.
12. Leppin AL, Bora PR, Tilburt JC, Gionfriddo MR, Zeballos-Palacios C, Dulohery MM, et al. The efficacy of resiliency training programs: a systematic review and meta analysis of randomized trials. PLoS One. 2014;9(10): e111420. doi: 10.1371/journal.pone.0111420

13. Windle G, Bennett KM, Noyes J. A methodological review of resilience measurement scales. Health Qual Life Outcomes. 2011;9(1): 8. doi: 10.1186/1477-7525-9-8
14. Min JA, Yoon S, Lee CU, Chae JH, Lee C, Song KY, et al. Psychological resilience contributes to low emotional distress in cancer patients. Support Care Cancer. 2013;21(9): 2469-76. doi: 10.1007/s00520-013-1807-6
15. Rozanski A, Blumenthal J, Kaplan, J. Impact of psychosocial factors on the pathogenesis of cardiovascular disease and implications for therapy. Circulation. 1999;99: 2192–2217.
16. Strike PC, Steptoe A. Psychosocial factors in the development of coronary artery disease. Prog Cardiovasc Dis. 2004;46: 337-47.
17. Terrill AL, Molton IR, Ehde DM, Amtmann D, Bombardier CH, Smith AE, et al. Resilience, age, and perceived symptoms in persons with long-term physical disabilities. J Health Psychol. 2016;21: 640-649. doi: 10.1177/1359105314532973
18. Kim GM, Lim JY, Kim EJ, Park SM. Resilience of patients with chronic diseases: A systematic review. Health Soc Care Community. 2019;27(4):797-807. doi: 10.1111/hsc.12620.
19. Loprinzi CE, Prasad K, Schroeder DR, Sood A. (2011). Stress Management and Resilience Training (SMART) Program to decrease stress and enhance resilience among breast cancer survivors: A pilot randomized clinical trial. Clin Breast Cancer. 2011;11: 364–368. doi: 10.1016/j.clbc.2011.06.008
20. Ye ZJ, Liang MZ, Qiu HZ, Liu ML, Hu GY, Zhu YF, et al. Effect of a multidiscipline mentor-based program, Be Resilient to Breast Cancer (BRBC), on female breast cancer survivors in mainland China—A randomized, controlled, theoretically-derived intervention trial. Breast Cancer Res Treat. 2016;158: 509–522. doi: 10.1007/s10549-016-3881-1
21. Sansom Daly UM, Wakefield CE, Bryant RA, Ellis S, Doolan E, Cohn RJ. Adapting evidence based psychological therapy to the computer screen for adolescent and young adult cancer survivors: Preliminary results from the ‘recapture life’ randomized controlled trial. Asia Pac J Clin Oncol. 2014;10: 36. doi: 10.1089/jayao.2015.0061
22. Kovacs AH, Bandyopadhyay M, Grace SL, Kentner AC, Nolan RP, Silversides CK, et al. Adult congenital heart disease coping and resilience (ACHD-CARE): Rationale and methodology of a pilot randomized controlled trial. Contemp Clin Trials. 2015;45: 385–393. doi: 10.1016/j. cct.2015.11.002
23. Bradshaw BG, Richardson GE, Kumpfer K, Carlson J, Stanchfield J, Overall J, et al. Determining the efficacy of a resiliency training approach in adults with type 2 diabetes. Diabetes Educ. 2007;33; 650–659. doi: 10.1177/0145721707303809
24. Vranceanu AM, Riklin E, Merker VL, Macklin EA, Park ER, Plotkin SR. Mind-body therapy via videoconferencing in patients with neurofibromatosis. Neurology. 2016;87: 806–814. doi: 10.1212/wnl.0000000000003005
25. Forbes S, Fikretoglu D. Building resilience: The conceptual basis and research evidence for resilience training programs. Rev Gen Psychol. 2018;22(4): 452-468 doi: 10.1037/gpr0000152
26. Burton NW, Pakenham KI, Brown WJ. Evaluating the effectiveness of psychosocial resilience training for heart health, and the added value of promoting physical activity: a cluster randomized trial of the READY program. BMC Public Health. 2009;9: 427. doi: 10.1186/1471-2458-9-427.

27. Burton NW, Pakenham KI, Brown WJ. Feasibility and effectiveness of psychosocial resilience training: a pilot study of the READY program. Psychol Health Med. 2010;15: 266-77. doi: 10.1080/13548501003758710.
28. Hawkes AL, Chambers SK, Pakenham KI, Patrao TA, Baade PD, Lynch BM, et al. Effects of a telephone-delivered multiple health behavior change intervention (CanChange) on health and behavioral outcomes in survivors of colorectal cancer: a randomized controlled trial. J Clin Oncol. 2013;31(18): 2313-21. doi: 10.1200/JCO.2012.45.5873.
29. Ryan A, Pakenham KI, Burton N. A pilot evaluation of a group acceptance and commitment therapy informed resilience training program for people with diabetes. Aust Psychol. 2019;1–12. doi: 10.1111/ap.12429.
30. Pakenham KI, Mawdsley M, Brown FL, Burton NW. Pilot evaluation of a resilience training program for people with multiple sclerosis. Rehabil Psychol. 2018;63(1): 29-42. doi: 10.1037/rep0000167.
31. Hayes SC, Luoma JB, Bond FW, Masuda A, Lillis J. Acceptance and commitment therapy: Model, processes and outcomes. Behav Res Ther. 2006;44: 1-25.
32. Dindo L, Van Liew JR, Arch JJ Acceptance and Commitment Therapy: A Transdiagnostic Behavioral Intervention for Mental Health and Medical Conditions. Neurotherapeutics. 2017;14(3): 546-553. doi: 10.1007/s13311-017-0521-3.
33. Graham CD, Gouick J, Krahé C, Gillanders D. A systematic review of the use of Acceptance and Commitment Therapy (ACT) in chronic disease and long-term conditions. Clin Psychol Rev. 2016;46: 46-58. doi: 10.1016/j.cpr.2016.04.009.
34. Spinhoven P, Drost J, de Rooij M, van Hemert AM, Penninx BW. Is experiential avoidance a mediating, moderating, independent, overlapping, or proxy risk factor in the onset, relapse and maintenance of depressive disorders? Cogn Ther Res. 2016;40(2): 150–163. doi: 10.1007/s10608-015-9747-8.
35. Kashdan TB, Rottenberg J. Psychological flexibility as a fundamental aspect of health. Clin Psychol Rev. 2010;30: 865-78.
36. Craig P, Dieppe P, Macintyre S, Michie S, Nazareth I, Petticrew M; Medical Research Council Guidance. Developing and evaluating complex interventions: the new Medical Research Council guidance. BMJ. 2008;337: a1655
37. Schardt C, Adams MB, Owens T, Keitz S, Fontelo P. Utilization of the PICO framework to improve searching PubMed for clinical questions. BMC Med Inform Decis Mak. 2007;7:16. doi: 10.1186/1472-6947-7-1
38. Chmitorz A, Kunzler A, Helmreich I, Tüscher O, Kalisch R, Kubiak T, Wessa M, Lieb K. Intervention studies to foster resilience - A systematic review and proposal for a resilience framework in future intervention studies. Clin Psychol Rev. 2018 Feb;59:78-100. doi: 10.1016/j.cpr.2017.11.002.
39. Connor KM, Davidson JRT. Development of a new resilience scale: The Connor-Davidson resilience scale (CD-RISC). Depress and Anxiety. 2003;18: 76-82.
40. Zigmond AS, Snaith RP. The hospital anxiety and depression scale. Acta Psychiatr Scand. 1983;67: 361–370. doi: 10.1111/j.1600-0447.1983.tb09716.x.

41. Crawford JR, Henry JD. The positive and negative affect schedule (PANAS): construct validity, measurement properties and normative data in a large non-clinical sample. Br J Clin Psychol. 2004 Sep;43(Pt 3):245-65.
42. Vickrey BG, Hays RD, Harooni R, Myers LW, Ellison GW. A health-related quality of life measure for multiple sclerosis. Qual Life Res. 1995;4: 187–206.
43. Johnson JA, Coons SJ, Ergo A, et al. Valuation of EuroQOL (EQ-5D) health states in an adult US sample. Pharmacoeconomics 1998; 13: 421–433.
44. Lamers S, Westerhof GJ, Bohlmeijer ET, ten Klooster PM, Keyes CL. Evaluating the psychometric properties of the mental health continuum-short form (MHC-SF). J Clin Psych. 2011;67:99-110.
45. Francis AW, Dawson DL, Golijani-Moghaddam N. The development and validation of the Comprehensive assessment of Acceptance and Commitment Therapy processes (CompACT). J Contextual Behav Sci. 2016;5(3): 134–145.
46. Grant S, Mayo-Wilson E, Montgomery P. CONSORT-SPI 2018 Explanation and Elaboration: guidance for reporting social and psychological intervention trials. Trials 2018; 19:406 https://doi.org/10.1186/s13063-018-2735-z
47. Calvert M, Kyte D, Mercieca-Bebber R, Slade A, Chan A-W, King MT. Guidelines for Inclusion of Patient-Reported Outcomes in Clinical Trial Protocols: The SPIRIT-PRO Extension. Jama 2018;319(5):483–494.
48. Polman CH, Reingold SC, Banwell B, Clanet M, Cohen JA, Filippi M, Fujihara K, Havrdova E, Hutchinson M, Kappos L, Lublin FD, Montalban X, O'Connor P, Sandberg-Wollheim M, Thompson AJ, Waubant E, Weinshenker B, Wolinsky JS. Diagnostic criteria for multiple sclerosis: 2010 revisions to the McDonald criteria. Ann Neurol.2011;69:292–302
49. Ivers NM, Halperin IJ, Barnsley J, Grimshaw JM, Shah BR, Tu K, Upshur R, Zwarenstein M.Allocation techniques for balance at baseline in cluster randomized trials: a methodological review. Trials. 2012 Aug 1;13:120. doi: 10.1186/1745-6215-13-120.
50. De Chirico G. Corso di training autogeno. Per superare ansie, paure, disturbi psicosomatici e per smettere di fumare. Red Edizioni. 2010.
51. O'Neill L, Latchforda G, McCrackenc LM, Grahama CD. The development of the Acceptance and Commitment Therapy Fidelity Measure (ACT-FM): A delphi study and field test. Journal of Contextual Behavioral Science. 2019;14:111-118.
52. Costantini M, Musso M, Viterbori P, Bonci F, Del Mastro L, Garrone O, et al. Detecting psychological distress in cancer patients: validity of the Italian version of the Hospital Anxiety and Depression Scale. Support Care Cancer. 1999;7: 121-7.
53. Terracciano A, McCrae RR, Costa PT Jr.Factorial and construct validity of the Italian Positive and Negative Affect Schedule (PANAS). Eur J Psychol Assess. 2003;19(2):131-141.
54. Solari A, Filippini G, Mendozzi L, Ghezzi A, Cifani S, Barbieri E, et al. Validation of Italian multiple sclerosis quality of life 54 questionnaire. J Neurol Neurosurg Psychiatry 1999;67: 158–62.
55. Balestroni G, Bertolotti G. EuroQol-5D (EQ-5D): an instrument for measuring quality of life. Monaldi Arch Chest Dis 2012; 78: 155-159

56. Petrillo G, Capone V, Caso D, Keyes CLM. The Mental Health Continuum–Short Form (MHC–SF) as a Measure of Well-Being in the Italian Context. Soc Indic Res (2015) 121:291–312 DOI 10.1007/s11205-014-0629-3
57. Giordano A, Pucci E, Naldi P, Mendozzi L, Milanese C, Tronci F, et al. Responsiveness of patient reported outcome measures in multiple sclerosis relapses: the REMS study. J Neurol Neurosurg Psychiatry 2009;80(9): 1023-8. doi: 10.1136/jnnp.2008.171181.
58. Kurtzke JF. Rating neurologic impairment in multiple sclerosis: an expanded disability status scale (EDSS). Neurology. 1983;33: 1444–52.
59. Donner A, Klar N. Statistical considerations in the design and analysis of community intervention trials. J Clin Epidemiol. 1996;49(4):435–439.
60. Leyrat C, Morgan KE, Leurent B, Kahan BC. Cluster randomized trials with a small number of clusters: which analyses should be used?. Int J Epidemiol. 2018;47(1):321–331. doi:10.1093/ije/dyx169
61. Landis JR, Koch GG. The measurement of observer agreement for categorical data. Biometrics 1977;33:159–74.
62. Shrout PE, Fleiss JL. Intraclass correlations: uses in assessing rater reliability. Psychol Bull 1979;86:420–8.
63. Bland JM, Altman DG. Statistical methods for assessing agreement between two methods of clinical measurement. Lancet 1986;1:307–17.
64. Huque H, Moreno-Betancur M, Quartagno M, Simpson JA, Carlin JB, Lee KJ. Multiple imputation methods for handling incomplete longitudinal and clustered data where the target analysis is a linear mixed effects model. Biometrical Journal. 2020;1–23. DOI: 10.1002/bimj.201900051
65. Pakenham KI, Landi G, Boccolini G, Furlani A, Grandi S, Tossani E. The moderating roles of psychological flexibility and inflexibility on the mental health impacts of COVID-19 pandemic and lockdown in Italy. J Contextual Behav Sci. 2020;17:109-118. doi:10.1016/j.jcbs.2020.07.003
66. Qiu J, Shen B, Zhao M, Wang Z, Xie B, Xu Y. A nationwide survey of psychological distress among Chinese people in the COVID-19 epidemic: implications and policy recommendations [published correction appears in Gen Psychiatr. 2020 Apr 27;33(2):e100213corr1]. Gen Psychiatr. 2020;33(2):e100213. Published 2020 Mar 6. doi:10.1136/gpsych-2020-100213

15. GLOSSARY OF ABBREVIATIONS

ACT	Acceptance and Commitment Therapy
CD-RISC 25	Connor-Davidson Resilience Scale 25
CI	Confidence Interval
CompACT	Comprehensive assessment Acceptance and Commitment Therapy processes
CPDI	The COVID-19 Peritraumatic Distress scale
DMAC	Data Management and Analysis Committee
EC	Ethics Committee
EQ-5D-3L	European Quality of life Five Dimensions
GCP	Good Clinical Practice

HADS	Hospital Anxiety and Depression Scale
ICC	Intraclass Correlation Coefficient
IDSMC	Independent Data and Safety Monitoring Committee
MHC	Mental Health Component
MHC-SF	Short form of the Mental Health Continuum
MRC	Medical Research Council
MS	Multiple sclerosis
MSQOL-54	The 54-items MS Quality of Life inventory
PANAS	Positive and Negative Affect Schedule
PHC	Physical Health Component
PI	Principal Investigator
PROMs	Patient reported outcome measures
PwMS	Persons with MS
QoL	Quality of life
RCT	Randomized controlled trial
READY	REsilience and Activities for every DaY
SD	Standard Deviation
TSC	Trial Steering Committee

16. LIST OF APPENDICES

S1 Appendix - Multi_READY for MS flowchart
S2 Appendix - READY for MS sessions
S3 Appendix - Relaxation sessions
S4 Appendix - READY for MS Session Fidelity Checklist
S5 Appendix - Relaxation Session Fidelity Checklist
S6 Appendix – Multi_READY for MS GANTT
